# Endocrine Manifestations in POEMS Syndrome: a case report and literature review

**DOI:** 10.1186/s12902-019-0355-6

**Published:** 2019-03-22

**Authors:** Hui Li, Yumeng Huang, Yan Li, Baozhong Zheng, Jingqiu Cui, Ming Liu

**Affiliations:** Department of Endocrine and Metabolism, Tianjin Medical University General Hospital, No.154, AnShan Road, HePing District, Tianjin, 300052 China

**Keywords:** POEMS syndrome, Endocrinopathy, Hyperpigmentation, Polyneuropathy, Plasmacytoma

## Abstract

**Background:**

POEMS syndrome, a rare systemic disease, is characterized by 5 components: Peripheral neuropathy, Organomegaly, Endocrinopathy, M protein elevation, and Skin changes. It usually presents with multiplex endocrine manifestations and is easily misdiagnosed and incorrectly treated.

**Case presentation:**

We report herein a case of POEMS syndrome that initially presented as hyperpigmentation and severe pitting edema of the lower extremities. Throughout the patient’s multiple hospitalizations for more than one year, he was treated erroneously for Addison’s disease and primary hypothyroidism due to the presence of limb numbness and weight loss. In addition, he was misdiagnosed with diabetic peripheral neuropathy due to a history of type 2 diabetes mellitus.

**Conclusion:**

Endocrinopathy is a critical feature of POEMS syndrome but its mechanisms are still poorly understood. The most common endocrine abnormality is hypogonadism, and the second is adrenal insufficiency, followed by hypothyroidism and subclinical hypothyroidism, then diabetes or glucose intolerance. In cases of the coexistence of endocrinopathy and unexplained peripheral neuropathy, especially in multisystem disorders, POEMS syndrome should be considered. Endocrine evaluation including thyrotropin, free thyroxine, fasting glucose, gonadal hormones, prolactin, cortisol, ACTH, and calcium should be assessed. The purpose of the current report was to provide increased awareness of POEMS syndrome.

## Background

POEMS syndrome [1], also known as Takatsuki syndrome [2], Crow-Fukase syndrome, or osteosclerotic myeloma, was first reported in 1938 by Scheinker [3]. It is a rare multisystem disease caused by an underlying plasma cell disorder. It is still not well understood. Endocrine manifestations are heterogeneous and may present as hypothyroidism, hypogonadism, and adrenal insufficiency [4]. The onset of endocrinopathy in POEMS syndrome has been observed in 67–84% of patients [5].

The term POEMS comes from its five hallmark elements: Polyneuropathy, Organomegaly, Endocrinopathy, M protein elevation, and Skin changes. Many patients are misdiagnosed with primary endocrine diseases such as adrenocortical hypofunction and hypothyroidism due to the syndrome’s complex endocrine manifestations. It was previously thought to be more common in Japanese progeny, but with increasing knowledge, large series have been observed in Europe, Africa, China, and India [6]. Its prevalence is 0.3 per 100,000 according to a national survey in Japan in 2003 [7].

## Case presentation

**Fig. 1:**
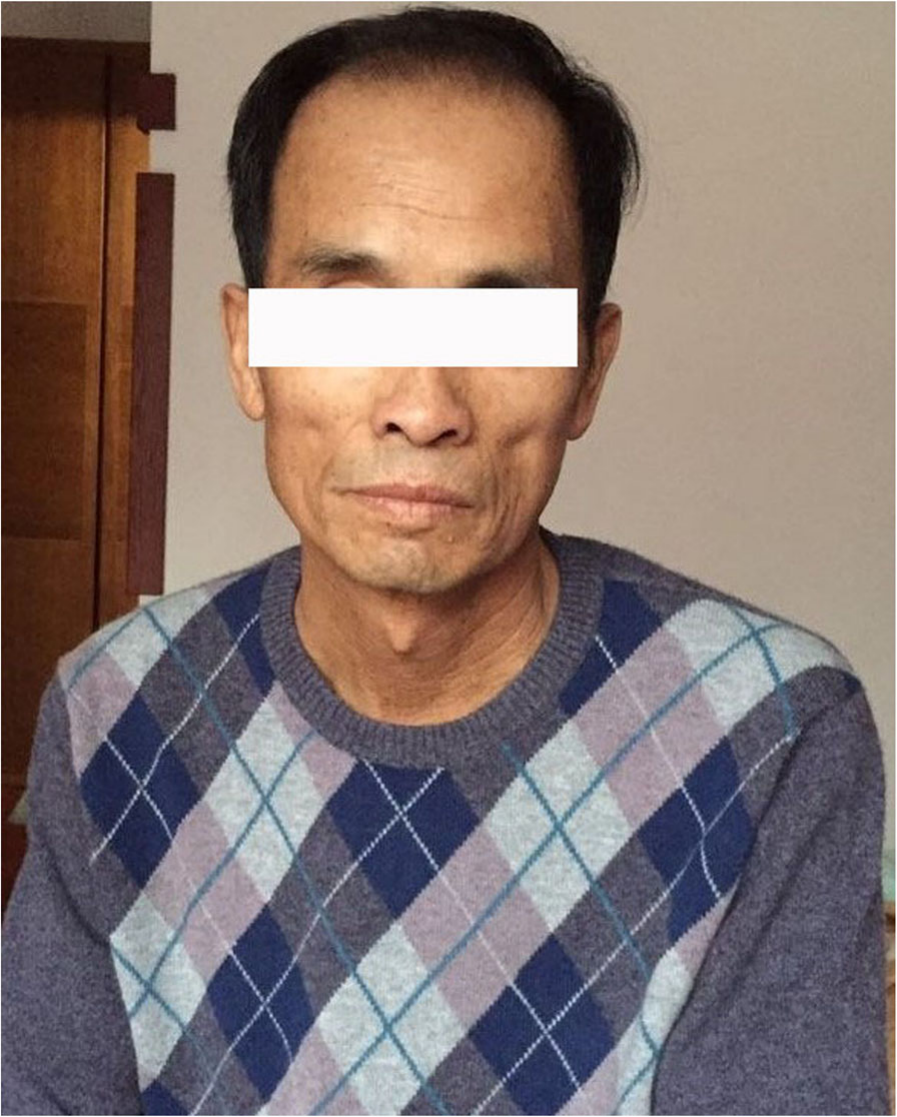
Before diagnosis, misery and depression were visible from the patient’s face

A 61-year-old male patient was admitted to our hospital with a prior one-year history of cutaneous hyperpigmentation. Loss of appetite, abdominal distension, constipation, dry skin, less sweat, and insomnia were concomitant symptoms. Ten months prior to admission, his symptoms became severe and were accompanied by symmetrical pitting edema, lower extremity numbness, and weakness in the left lower limbs. Brain MRI showed cerebral infarction, and the patient was treated appropriately. One month later, he was diagnosed with hypothyroidism and Addison’s disease (AD) for severe edema of the lower extremities, unexplained cutaneous pigmentation, and higher ACTH levels (Tables 1 and 2). Hydrocortisone 20 mg and Levothyroxine 12.5 μg per day as well as diuretic therapy were administered, and the symptoms mildly improved. After discharge from the hospital, he gradually stopped the diuretic drugs and the doses were adjusted to hydrocortisone 40 mg and Levothyroxine 200 μg per day based on the lab tests. Concomitantly, he experienced pain and numbness in his lower limbs. Since the onset of illness, his general condition was poor. The patient suffered from decreased appetite, poor sleep, weight loss of 15 kg, and hyposthenia (Fig. 1).

**Table 1:** Thyroid function of the patient

Date	FT3 (3.8–6.0 pmol/L)^a^ (3.5–6.5 pmol/L)^b^	FT4 (7.86–14.41 pmol/L)^a^ (11.5–23.5 pmol/L)^b^	TSH (0.34–5.6 mIU/mL)^a^ (0.3–5.0 mIU/mL)^b^	TGAb (0–40 IU/mL)	TPOAb (0–35 IU/mL)
1st day^a^	**2.88**	9.73	**10.520**	< 20	< 10
23rd day^a^	**3.67**	9.25	**9.889**	–	–
8th month^a^	**2.23**	12.85	1.557	–	–
9th month^b^	4.17	**10.20**	3.84	–	–
10th month^b^	4.79	**8.97**	3.11	–	–
12th month^b,c^	5.18	18.52	0.11	–	–

^a^Reference range of one hospital, ^b^reference range of another hospital, ^c^ after lenalidomide was administered, boldface type indicate values out of range

**Table 2:** Adrenal function of the patient

Date	Cor (6.7–22.6 μg/dL)	ACTH (0–46 pg/ml)	24 h urine Cor (30–110 μg/24 h)
1st day	21.6	**67.4** ^**a**^	49.72
8th month	15.8	**180.0** ^**b**^	246.25
9th month	11.4	**–**	–
10th month	10.65	**–**	–
12th month	11.51	–	–

^a^Elevated level of ACTH, considered relatively adrenal insufficiency, ^b^After hydrocortisone was administered, the ACTH level was elevated, boldface type indicate values out of range

The patient’s past history showed he was a carrier of hepatitis B virus for 60 years, psoriasis for 40 years with external steroid use, had a 10-year history of type 2 diabetes mellitus controlled by insulin glargine and voglibose, and had hypertension for 2 months. In addition, he was diagnosed with depression two months prior and was treated with flupentixol and melitracen tablets without obvious improvement.

**Fig. 2:**
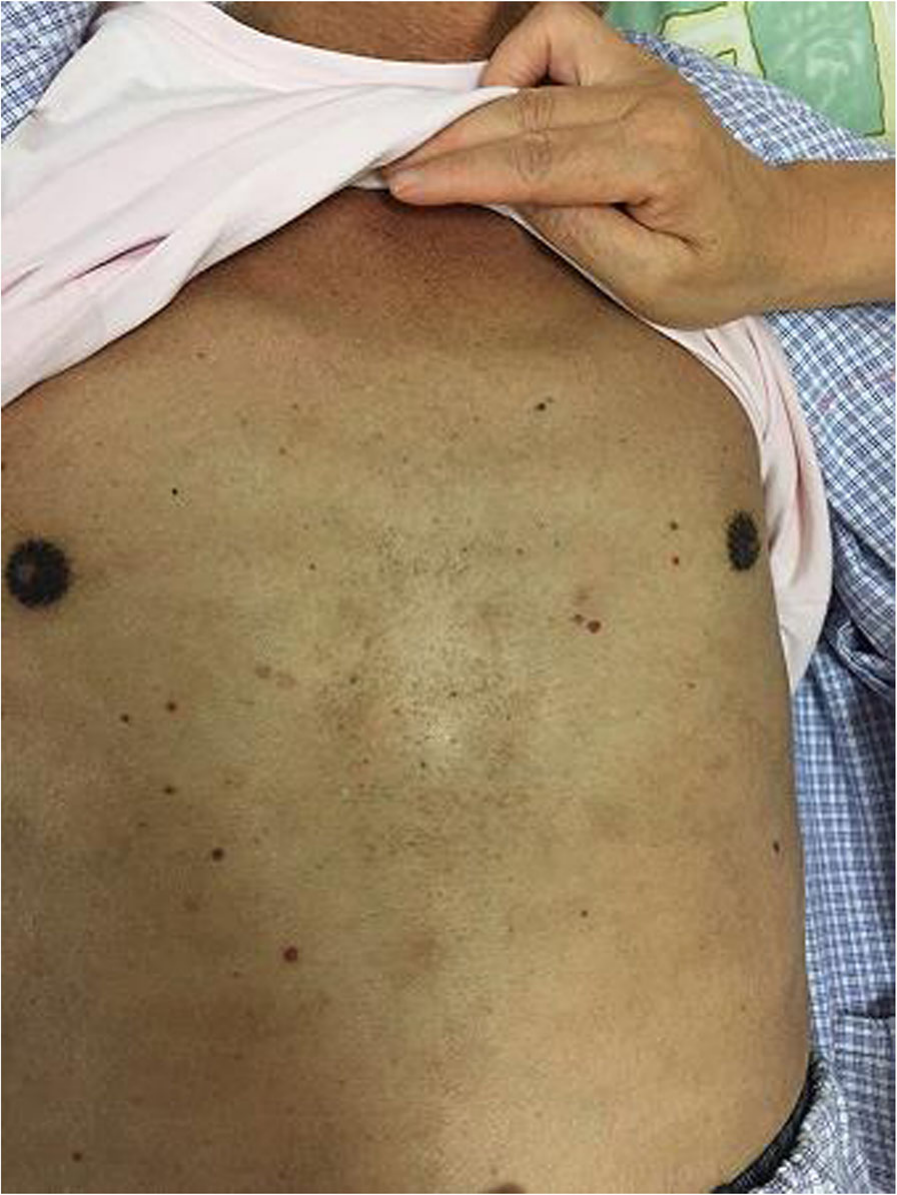
Cutaneous pigmentation

**Fig. 3:**
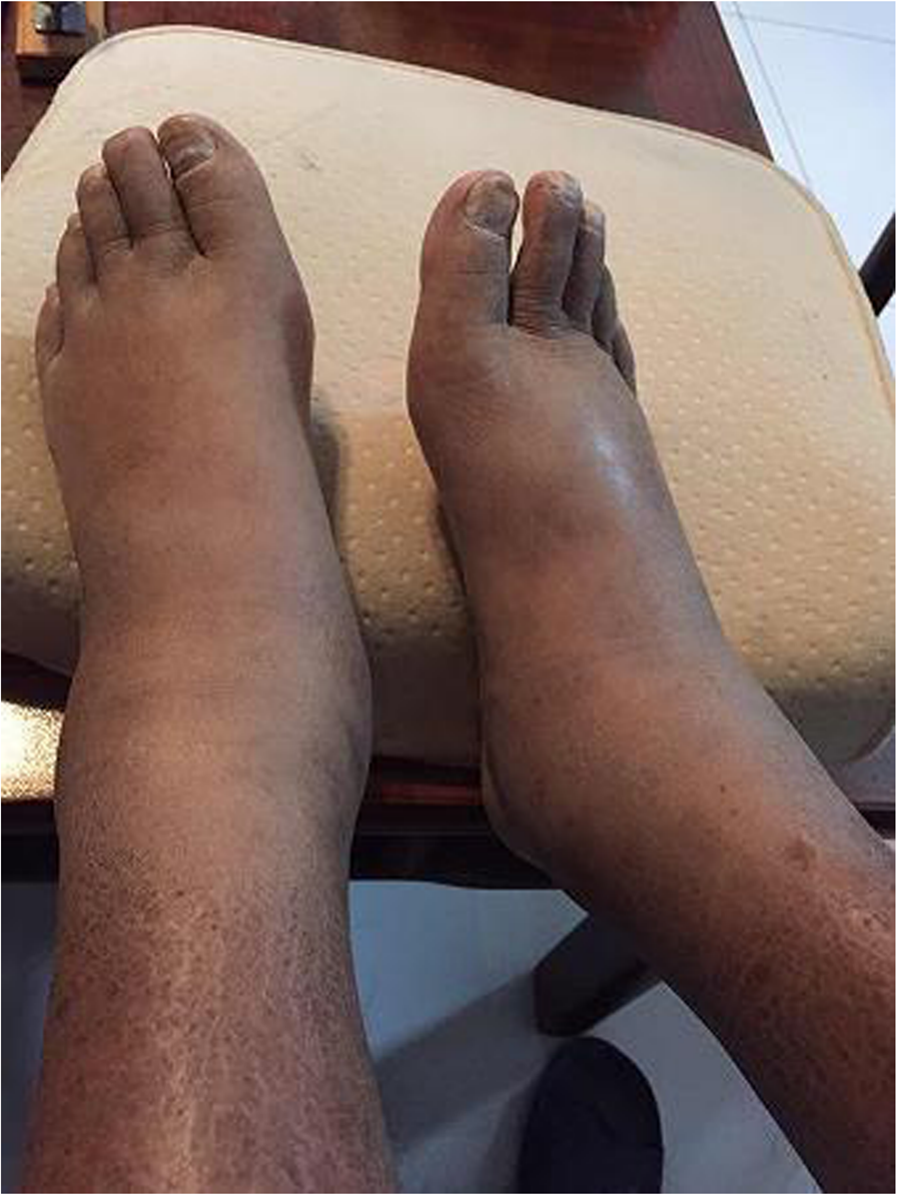
Symmetrical edema of the double limbs

**Fig. 4:**
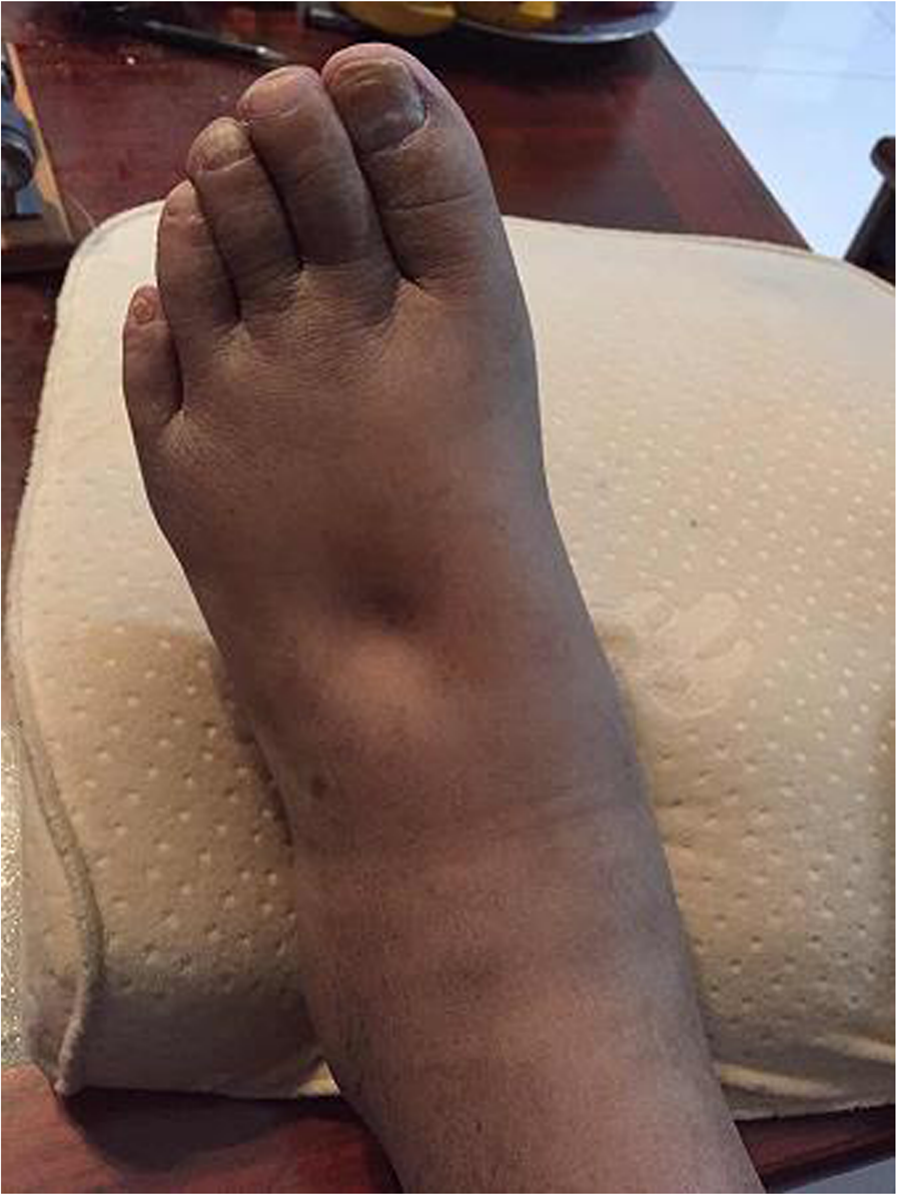
Severe edema of the left foot

**Fig. 5:**
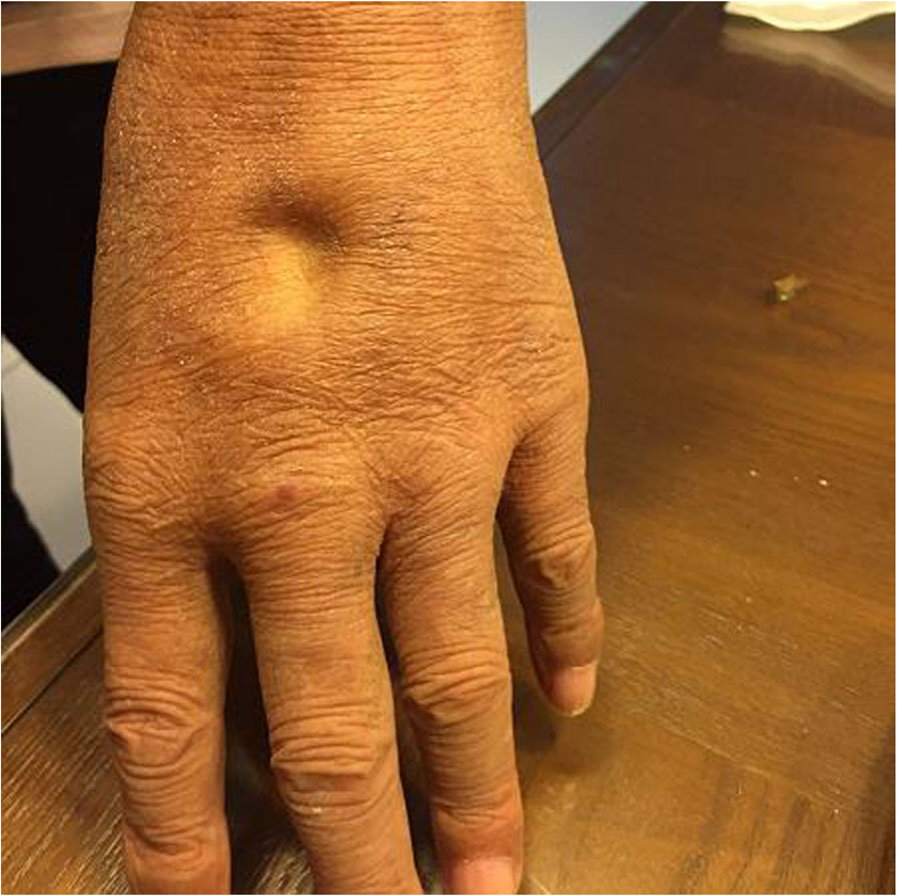
Edema of the back of the hand

**Fig. 6:**
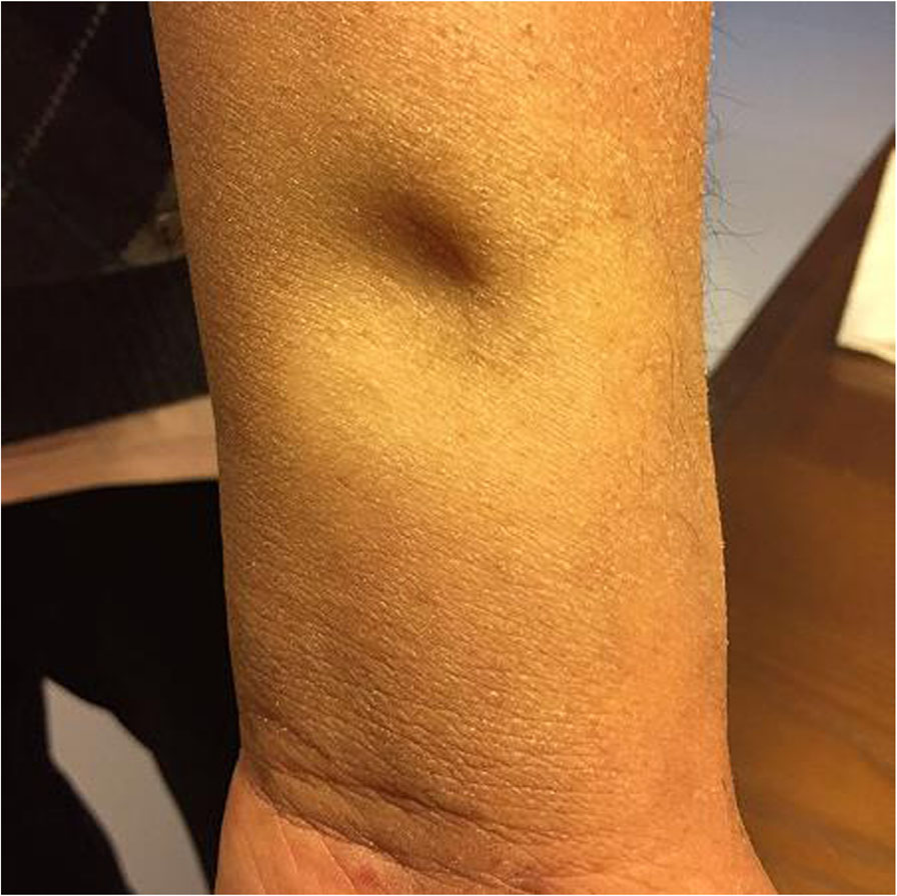
Edema of the upper limbs

Examination showed T 36.1°C, P 75 bpm, R 16 tpm, BP 140/85 mmHg, H 176 cm, W 62 kg, and BMI 20 kg/m^2^. The patient had diffuse cutaneous pigmentation of his skin and mucous membranes, especially the areolas and armpits. His superficial lymph nodes were not palpable. He had edema of the eyelids and pitting edema of the upper and lower extremities (Figs. 2, 3, 4, 5, 6). Neurological examination showed grade IV muscle strength in his left lower limbs. His physiological reflexes were normal, and his pathological reflexes were not elicited. His thyroid function and adrenal function were abnormal (Tables 1 and 2).

Mild anemia was noted, with HBsAg(+), HBsAb(−), HBeAg(−), HBeAb(+), and HBcAb(+). Rheumatoid factor, anti-nuclear factor, and anti-HIV were negative. Serum electrolytes revealed normal sodium levels. BNP was 2010 ng/L (450–900), and TNI < 0.012 ng/ml (0–0.12). Albumin was 34 g/L (35–55), and fasting blood glucose (FBG) was 5.3–6.5 mmol/L (3.6–5.8). Electroneuromyography disclosed polyneuropathy of the upper and lower extremities. The results of an adrenal CT scan and a head MRI were negative. Abdomen B-ultrasound presented splenomegaly (4.8 × 13.8 cm) and ascites. UCG indicated pericardial effusion with 59% EF. Bone marrow aspirate showed medullary phagocytosis. No M protein was found via serous protein electrophoresis but an immunofixation examination revealed a monoclonal IgAλ peak. The diagnosis POEMS syndrome was made.

**Fig. 7:**
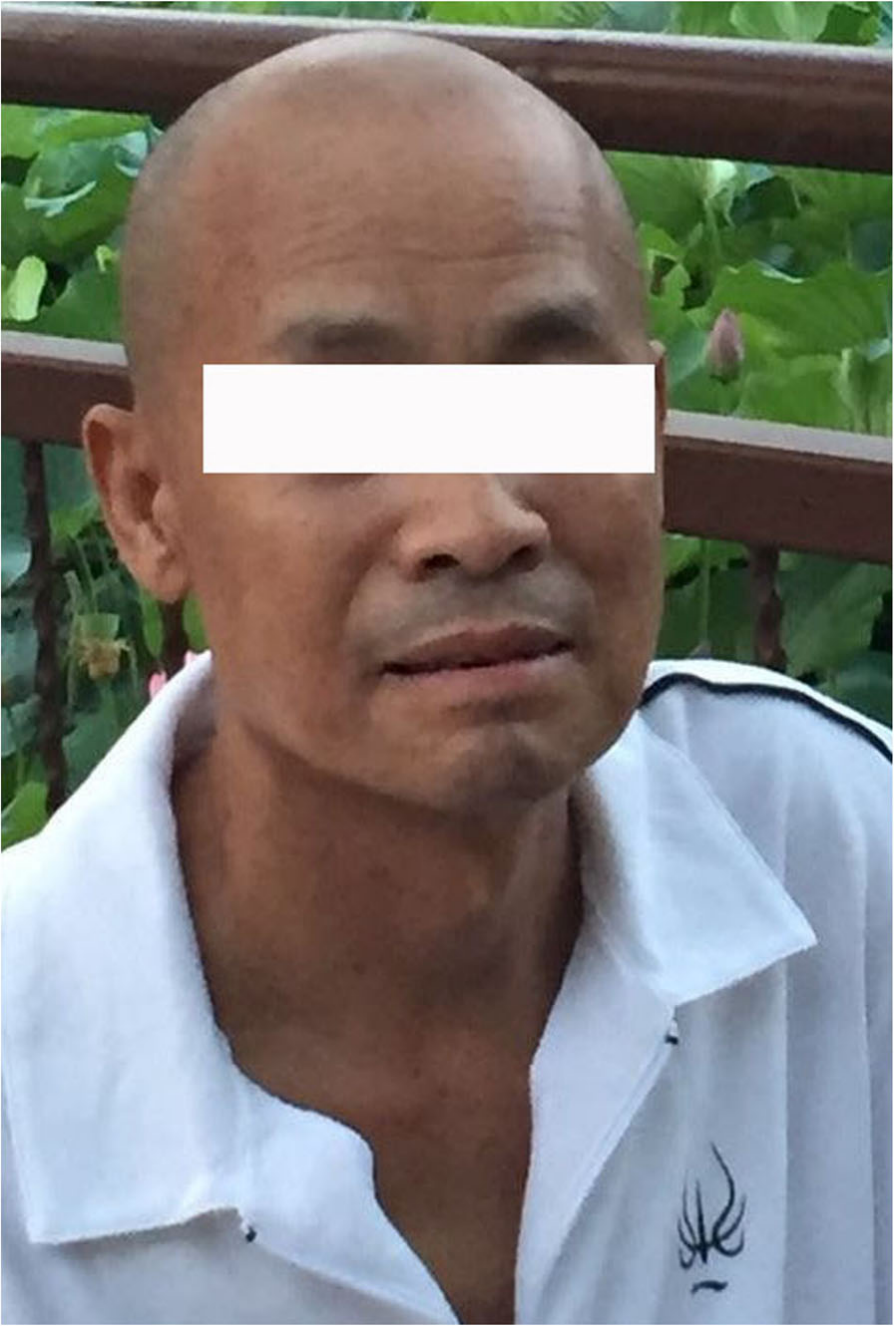
After ASCT treatment, the patient gained weight

The patient was initially treated with a very expensive drug, Revlimid (lenalidomide capsules 25 mg), which is generally used for multiple myeloma (MM) patients. He then underwent autologous stem cell transplantation (ASCT) and experienced significant improvement of his hyperpigmentation and weight loss(Fig. 7). Hydrocortisone and Levothyroxine were gradually decreased. His diabetes was also relieved.

## Discussion

The acronym POEMS was coined by Bardwick et al. in 1980 to describe a rare complex syndrome characterized by polyneuropathy, organomegaly, endocrinopathy, M protein, and skin changes [8]. Essentially, it is a paraneoplastic syndrome caused by a proliferative monoclonal plasmacyte attack with an indolent course. In other words, it is a disease of the blood system, but the etiology of POEMS syndrome currently remains unknown. The increased production of proangiogenic and proinflammatory cytokines such as interleukin-1β (IL-1β), IL-6, IL-12, and tumor necrosis factor-α (TNF-α), especially vascular endothelial growth factor 165 (VEGF165), are the critical elements in its pathogenesis. VEGF, secreted by the plasmacytes, is known to target the endothelial cells and is blamed for ascites, edema, organomegaly, pleural effusion, and neuropathy [9–11].

POEMS syndrome is more common in males in their 50s and 60s. According to the diagnostic criteria published by Despenzieri et al. at the Mayo Clinic based on clinical and laboratory features in 2003 [1]. The diagnosis of POEMS syndrome requires the presence of three major associations, two of which must include polyradiculoneuropathy and clonal plasma cell disorder, and at least one minor criterion. In 2014, this was confirmed when both of the mandatory major criteria (polyneuropathy and monoclonal plasma cell proliferative disorder), one of the three other major criteria (Castleman disease, sclerotic bone lesions, and VEGF elevation), and one of the six minor criteria (organomegaly, extravascular volume overload, endocrinopathy, skin changes, papilledema, and thrombocytosis/polycythemia) were present [6]. Our patient fulfilled the 2003 criteria but failed to meet the 2014 criteria for the absence of VEGF levels.

POEMS syndrome is a multisystem disease with a wide spectrum of clinical manifestations and progressive features, which makes its diagnosis a challenge and easily leads to misdiagnosis. Cui et al. reported 6 patients (30%) who were misdiagnosed with primary endocrine diseases out of 20 patients in China in 2012. Our patient visited four different hospitals and was hospitalized six times within one year of the first appearance of his symptoms. The diagnoses included hypothyroidism, chronic adrenal insufficiency (Addison’s disease), type 2 diabetes mellitus, cerebral infarction, heart failure, and depression, among other conditions. Questions were raised concerning his lack of improvement although his thyroid function and cortisol levels returned to normal ranges after replacement therapy. His experience caused great mental and economic pressures to himself and his family, similar to the Southern Song Dynasty poem: “After endless mountains and rivers that leave doubt whether there is a path out, suddenly one encounters the shade of a willow, bright flowers, and a lovely village.” The patient was finally diagnosed correctly and treated. We should note that endocrinopathy manifests as a key part of POEMS syndrome; therefore, we reviewed the literature concerning endocrinopathy in this syndrome.

Endocrinopathy is a critical feature of POEMS syndrome but the mechanisms are still poorly understood. Among such patients, 67–84% had at least one recognized endocrinopathy based on large retrospective series [12–14]. Endocrine disorders including hypogonadism, hyperprolactinaemia, diabetes, and hypothyroidism have been reported in related studies.

Zong et al. investigated 14 patients in 2012 in China [15]. Ten suffered from endocrinopathy and all presented with hypothyroidism, except one with amenorrhea, two with impotence, one with Addison’s disease, and one with hypoparathyroidism. Dun also found a high prevalence of hypothyroidism in 24 cases [16]; 17 (70.8%) had recognized hypothyroidism (including 11 with clinical hypothyroidism and 6 with subclinical hypothyroidism). Hypothyroidism may be one of the causes of edema/effusions. According to another study [17], 5 of 8 patients had hypothyroidism, 1 had irregular menses, 7 had erectile dysfunction, and 4 had increased prolactin levels. However, according to studies from 1984 to 2017 (Table 3) [1, 4, 15, 18, 19], the most common endocrine abnormality was hypogonadism [20].

**Table 3:** Comparison of endocrinopathy in different series

Characteristics		Nakanishi et al.[15], *N* = 102 (1984) No.(%)of patients	Soubrier et al.,[18] *N* = 25 (1994) No.(%)of patients	Dispenzieri et al.,[1] *N* = 99 (2002) No.(%)of patients	Gandhi et al.,[4] *N* = 64 (2007) No.(%)of patients	Hu et al. [19], *N* = 23 (2017) No.(%)of patients
Any endocrine abnormality		NR	NR	66/99(66)	54/64(84)	22/23(96)
Adrenal axis abnormality		NR	NR	16/35(46)	6/9(67)	10/17(59)
Hyperprolactinemia		NR	4/17(24)	5/25(20)	10/35(29)	9/15(60)
Diabetes mellitus or glucose intolerance		26/93(28)	9/22(41)	3/99(3)	24/50(48)	5/23(22)
Hypothyroidism		5/21(24)	10/22(45)	26/99(26)	28/48(58)	13/22(59)
Hypocalcemia		NR	NR	1/4(25)	14/51(27)	3/10(30)
Gonad related dysfunction	Hypogonadism	NR	9/19(47)	55/99(55)	26/33(79)	7/18(39)
	Erectile dysfunction (males)	39/50(78)	13/13(100)	44/63(71)	23/38(61)	NR
	Gynecomastia or galactorrhea (males)	43/63(68)	10/13(77)	17/63(27)	10/38(26)	NR
Multiple endocrine abnormalities		NR	NR	NR	29/54(54)	NR

*Data are number (percentage) of patients, NR = not reported

Male patients tended to have erectile dysfunction and gynecomastia [21], while amenorrhea usually presented in female patients. Hyperprolactinemia was present in approximately one-quarter of patients. The second most common abnormality was adrenal insufficiency, followed by hypothyroidism and subclinical hypothyroidism, then diabetes or glucose intolerance. Additionally, 27–45% of patients suffered from hypocalcemia. More than half had multiple axes involvement, including gonadal, thyroid, pancreas, and adrenal disorders. Dispenzieri et al. found high morbidity of adrenal insufficiency (while elevated levels of ACTH might explain the symptom of hyperpigmentation) [4], which differed in previous studies. Our patient complained of hyperpigmentation at the onset of disease and was diagnosed with adrenal insufficiency but received no benefits from hydrocortisone therapy. However, he recovered after ASCT treatment and could move freely by himself with reduced endocrine drugs. It should be noted that great controversy exists among the studies concerning the prevalence of adrenal insufficiency, but the reason for these differences may due to incomplete medical records. In other words, appropriate laboratory tests were not ordered for some patients, leading to the high misdiagnosis rate of POEMS syndrome.

Peripheral neuropathy is the marked symptom of POEMS syndrome [22]. Because the characteristics of neuropathy are nonspecific, patients with diabetes are frequently misdiagnosed with diabetic peripheral neuropathy (DPN) when they present at endocrinology departments. Our patient was a typical example. He was treated for DPN with lipoic acid, mecobalamin, and other drugs, but with little effect. He et al. summarized the differences between the neuropathy of POEMS syndrome and DPN, noting that a longer process and slower progress are features of DPN with axonal degeneration alone, while there is a combination of axonal and demyelinating lesions in POEMS syndrome [23].

Why do endocrine abnormalities occur in POEMS syndrome? Monika et al. proposed that POEMS syndrome is an antibody-mediated immune disorder for specific antibody binding directly against hypophysis, which was found at autopsy in typical POEMS cases [24]. However, this could not explain the high incidence of primary hypothyroidism or elevated ACTH levels. Other studies found that the structure of the endocrine glands remained normal, indicating that there were functional endocrine abnormalities rather than structural changes [25, 26]. As we mentioned previously, VEGF is a critical factor in POEMS syndrome. Gandhi et al. hypothesized that it might affect some of the endocrine axes through disruption of the cytokine balance that regulates hormone secretion in the endocrine glands.

One study found that the median time from the onset of symptoms to diagnosis was 18 months in patients with POEMS syndrome. Thus, the purpose of the current report was to provide increased awareness of POEMS syndrome. In cases of the coexistence of endocrinopathy and unexplained peripheral neuropathy, especially with multisystem disorders, this syndrome should be taken into consideration. Endocrine evaluation including thyrotropin, free thyroxine, fasting glucose, gonadal hormones, prolactin, cortisol, ACTH, and calcium should be assessed.

According to a 2016 survey with 2273 person-years of follow-up, the 10-year overall survival (OS) was 62%. In a multivariate analysis, the three factors associated with superior OS were younger age, albumin greater than 3.2 g/dL, and attainment of complete hematologic response.

## Conclusion

In summary, POEMS syndrome is a rare multisystem disease with multiplex endocrine manifestations that can be easily misdiagnosed. Thorough inspections including the three endocrine axes (adrenal, thyroid, and gonad) and diabetes should be taken into consideration to distinguish it from diseases such as DPN, MM, and others.

## Abbreviations

ACTH: adrenocorticotropic hormone
AD: Addison’s disease
ASCT: autologous stem cell transplantation
BNP: brain natriuretic peptide
CT: computed tomography
DPN: diabetic peripheral neuropathy
EF: ejection fraction
FBG: fasting blood glucose
MM: multiple myeloma
MRI: magnetic resonance imaging
TNI: cardiac troponin I
UCG: ultrasound cardiogram
